# *Mens sana in corpore sano*: Does the Glycemic Index Have a Role to Play?

**DOI:** 10.3390/nu12102989

**Published:** 2020-09-29

**Authors:** Lionel Carneiro, Corinne Leloup

**Affiliations:** 1 Department of Biological Chemistry and Pharmacology, Ohio State University, Columbus, OH 43210, USA; 2 Centre des Sciences du Goût et de l’alimentation, UMR CNRS 6265, INRA 1324, AgroSup, Univ. Bourgogne Franche-Comté, F-21000 Dijon, France; corinne.leloup@u-bourgogne.fr

**Keywords:** cognition, nutrition, metabolism, neurodegeneration, ketone bodies, glycemia, nutrition therapy

## Abstract

Although diet interventions are mostly related to metabolic disorders, nowadays they are used in a wide variety of pathologies. From diabetes and obesity to cardiovascular diseases, to cancer or neurological disorders and stroke, nutritional recommendations are applied to almost all diseases. Among such disorders, metabolic disturbances and brain function and/or diseases have recently been shown to be linked. Indeed, numerous neurological functions are often associated with perturbations of whole-body energy homeostasis. In this regard, specific diets are used in various neurological conditions, such as epilepsy, stroke, or seizure recovery. In addition, Alzheimer’s disease and Autism Spectrum Disorders are also considered to be putatively improved by diet interventions. Glycemic index diets are a novel developed indicator expected to anticipate the changes in blood glucose induced by specific foods and how they can affect various physiological functions. Several results have provided indications of the efficiency of low-glycemic index diets in weight management and insulin sensitivity, but also cognitive function, epilepsy treatment, stroke, and neurodegenerative diseases. Overall, studies involving the glycemic index can provide new insights into the relationship between energy homeostasis regulation and brain function or related disorders. Therefore, in this review, we will summarize the main evidence on glycemic index involvement in brain mechanisms of energy homeostasis regulation.

## 1. Introduction

Nutrition has been part of the treatment employed for diabetes and obesity for decades. Therefore, specific food choices to help control weight and glucose homeostasis represent an important step in the establishment of a suitable diet. To assist patients, Jenkins et al. established the glycemic index (GI) concept in 1981 [1]. GI measures the impact of an individual food on the blood glucose level over time when compared to the effect of glucose itself (GI = 100). The glycemic response will thus depend on both the quantity and quality of carbohydrates (sugars, starch, or fibers) in the food. Consequently, a low-GI food (GI ≤ 55) contains high-quality carbohydrates and will not raise glycemia as much as a high-GI food (GI ≥ 70) for the same amount of carbohydrate. However, since the GI does not consider the amount of carbohydrate ingested, a glucose load (GL) value was developed. Thereby, GL represents a product of GI (quality of carbohydrate) and the quantity of carbohydrate ingested. Low GL is considered to be below 10, while high GL is above 20 when 10 g of glucose has a GL of 10 (Table 1).

**Figure 1:**
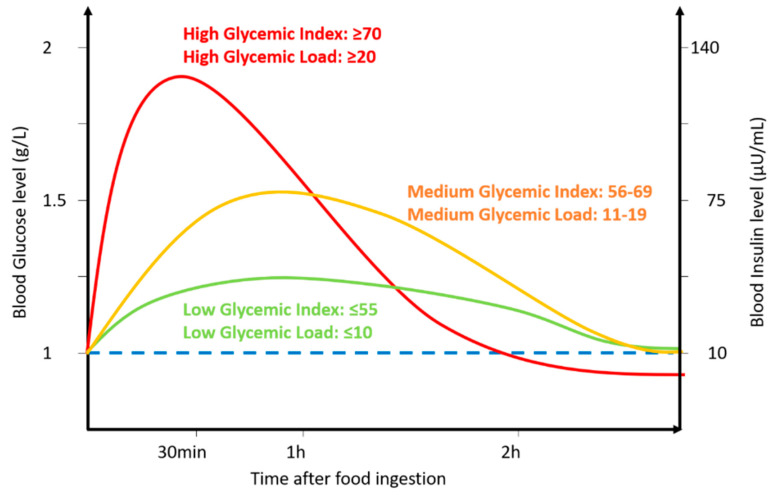
Schematic diagram of the influence of GI or GL on blood glucose (left axis) or insulin (right axis). Low vs. medium vs. high GI or GL and their corresponding value range are indicated.

In fact, low-GI foods are digested and absorbed slowly compared to high-GI foods. Therefore, low-GI foods induce a limited increase in blood glucose that lasts for an extended period. Conversely, high-GI foods are easily digested and absorbed, and induce a rapid and high increase in blood glucose, followed by insulin secretion that often leads to transient hypoglycemia (Figure 1) [2,3].

Diet interventions are used in physiological conditions, and are also used in sports for weight management, to lose fat mass or gain lean mass. In addition, metabolic disorders are commonly associated with nutritional recommendations to induce weight loss, control glucose levels, or manage dyslipidemia [4]. Most notably, specific diets have also been used in non-metabolic conditions for centuries. For instance, epilepsy treatment has included a ketogenic diet (KD) for almost 100 years [5]. Interestingly, specific diets are used in cancer, cognitive improvement, neurological disorders, and mental diseases [6,7,8,9]. Most of the diets involved are characterized by a low carbohydrate content or low-GI food. Although GI and GL are determined for individual foods, diets based on these values have been developed, as well as methods of evaluation of GI/GL meals [10,11]. Therefore, the diets used in medical nutrition therapies are expected to be low-GI diets. In fact, the impact of a meal on glycemia is likely to be a key factor in disease development. Indeed, since glucose represents the main source of energy for our body in a healthy state, a disturbed glucose supply will alter the normal function of cells.

The brain represents 2% of the body weight, while it is the main glucose consumer (20%) in humans [12]. Therefore, brain control of the glucose supply is finely regulated by the brain itself. Indeed, the brain possesses the ability to sense glucose levels and to trigger an adaptive response when glucose levels are low. Then, the brain will stimulate food intake or glucose production by peripheral organs. This will maintain a constant energy supply for brain activity [13]. Therefore, brain glucose detection is a key mechanism for both brain activity control and energy homeostasis regulation. The brain plays a central role in the maintenance of energy homeostasis of the whole body. Moreover, nutrient sensing is one of the most regulated mechanisms and involves specific regions such as the hypothalamus [14]. Among the nutrients sensed, glucose is the most studied and characterized [15]. Since the GI impacts the blood glucose level, the composition of meals based on GI is expected to involve the brain circuits of glucose sensing. Moreover, low-GI diets are also prone to an increased production of ketone bodies since they are low in carbohydrates [8]. Finally, the carbohydrate composition of a meal induces changes in gut microbiota and in signals involved in food intake regulation and neurological disorders [16]. Overall, by involving key signals of both metabolic regulations and neuronal functions, GI diets can improve cellular and whole-body metabolism via brain regulation. Indeed, recent reports demonstrate a clear relationship between brain function and energy homeostasis. For instance, Alzheimer’s disease (AD) shows metabolic defects, including glucose uptake deficiency in the brain, insulin resistance, and even food intake alterations [17,18]. Furthermore, neurodegeneration is also associated with metabolic impairment and diabetes [19,20].

Although low-GI diets have been developed to help diabetic people to manage their body weight and glycemia, numerous effects on brain function have also been described. Therefore, the cognitive function (memory, attention, etc.) in healthy people, as well as improvements in the brain function of patients with brain dysfunctions, have been measured in relation to diets. Autism Spectrum Disorders (ASD), epilepsy, neurological disorders, and seizures have all been tested and displayed interesting results.

Therefore, in this review, we aim to present the current understanding of the effect of diets and their GI/GL on brain functions, including cognition and energy homeostasis regulation. Furthermore, the mechanisms involved will be described. Indeed, common mechanisms in both cognition and energy sensing can provide new insights to develop novel therapeutic approaches in diseases associated with these functions. Finally, learning about the mechanisms involved should help us understand the relationship between metabolism and neurological function. The results presented here were obtained from PubMed and Web of Science research using combinations of the following key words: brain, neurodegenerative disease, energy homeostasis, cognition, glucose load, glycemic index, and nutrition. Such an understanding is of great importance for developing novel nutritional approaches for disease treatment, in addition to generating new nutritional recommendations for healthy people. 

## 2. Effect of a Glycemic Index Diet on Brain Function

Several studies have highlighted the role of a low-GI diet in insulin sensitivity, vascular system function, and weight management [21]. Recommendations in diabetic patients to help control their blood glucose level represent one of the most important applications of GI/GL indexes, despite some caveats in their interpretation. Besides this metabolic role, diets are used in neurodegenerative disorders, cancer, and even seizures. Such diet interventions started to gather interest following the discoveries of the influence of nutrients on brain function and notably on cognition, brain plasticity, and synaptic function, among others [9]. More recently, studies on the effects of specific diets on brain function gave rise to new evidence on the importance of nutrition in alterations and thus improvements. Low-GI diets have been used to ameliorate cognitive function, but also improve several pathological symptoms observed in specific neurological disorders, from dementia and depression, to ASD and AD (Table 2) [7].

Indeed, since glucose represents the main energy source for the brain, glucose level control appears critical for maintaining normal brain activity. Furthermore, neurological disorders are often associated with changes in neuronal activity, which can be targeted by modifying the availability of energetic substrates [22]. Nevertheless, diets can also be used in healthy people in a non-metabolic context. Indeed, different attempts to find out how to improve health through diet have been tested for decades in terms of physical activity, memory, and attention [23].

### Effect of the Glycemic Index on Cognitive Function in Healthy People

Normal life requires a balanced diet with adapted macro- and micronutrients to maintain optimal cellular functions. Among other factors, cognition is likely to be altered by diet due to the high energy needs of the brain. Furthermore, the poor feeding habits of modern societies can very much alter normal cognitive function in healthy people. Therefore, a healthy diet can benefit healthy people and unhealthy populations. Aging, for instance, is often accompanied by cognitive decline. Therefore, determining the effects of dietary habits on cognition could be important for delaying aging-related declines. In this regard, the study of cognition in healthy elderly populations has tried to determine the role of GI/GL.

A recent study revealed that a low-GL diet contributes to maintaining a better cognitive function in the elderly. This result, along with others, support the role of GI/GL during the aging process (Table 3) [24,25,26]. Furthermore, these studies show either a decreased risk of dementia or AD occurrence. These observations confirm a negative effect of Western diets on cognition, which has been previously documented [27,28,29]. However, the study of Garber et al. indicates that only people with poor glucose regulation display a positive effect of GL [30]. High fat associated with high GI has been shown to induce insulin resistance, while the same fat content with a low GI improved insulin sensitivity [31]. Therefore, here, it is possible that the effects observed are due to insulin sensitivity improvement. Indeed, insulin is known to participate in cognitive function [32]. Furthermore, the improvement of glucose homeostasis could improve the energy supply to the brain and thus cognitive function.

Overall, the results presented do not completely address a role in healthy people since the effects observed are those on low glycemic control people. In support of that, previous analyses performed in younger populations over the past years have failed to give a precise answer on the effect of GI/GL on cognition in healthy people. In fact, a meta-analysis and study conducted by Philipou et al. revealed discrepancies in the results obtained on the role of GI/GL in cognition in healthy persons [33,34]. Such observations make it difficult to draw a conclusion on the relationship between GI/GL and cognition in healthy people (Table 3).

Younger populations of schoolchildren could also be targeted by diet adjustments to improve learning and memory functions. Indeed, different studies on adolescents have described a positive relationship between the GI/GL of breakfast meals and cognition through improving learning, but also attention, stress, and even mood [35,36,37,38,39,40]. Here, schoolchildren were divided into low- or high-GI breakfasts or no breakfast at all. Then, cognitive tasks were used to test the ability of the diets to inhibit cognitive interference (Stroop test [41]), memory tasks, focus, learning and mood, hunger and thirst, and fatigue in adolescents.

It was previously shown that adolescents who consume breakfast exhibit improved cognitive function compared to those who do not consume breakfast [42]. The higher glucose supply was then expected to help maintain the better performance displayed in the cognitive tests. This result confirms previous results discussed in the elderly. Nonetheless, introducing low- and high-GI breakfast groups to a breakfast omission group provides a more precise picture on the putative mechanism involved. Thereby, both low- and high-GI breakfasts show improved adolescent cognition compared to the group without breakfast. Such a result supports the need of an energy supply for brain function. Moreover, low-GI breakfasts are more effective in these improvements than high GI breakfasts. This greater improvement related to low-GI breakfasts is also associated with lower glycemic and insulinemic responses. In fact, a high-GI breakfast group presented the lowest reaction time during the Stroop test. However, this increase in reaction was to the detriment of accuracy, which was significantly better in the low-GI group. Furthermore, this gain of accuracy was better maintained across the morning [38]. Finally, low-GI breakfast children display better results in cognitive tests assessing their working memory, as well as attention (Table 3).

All of these studies support the role of glucose in cognition improvement since both low- and high-GI breakfasts are beneficial compared to no breakfast. However, since a low-GI breakfast gives better results than a high-GI breakfast, the role of a high circulating glucose level in such an improvement is disputed [43]. Indeed, since a low-GI meal induces a lower increase in blood glucose compared to a high-GI meal, a high glucose level cannot be the main or only contributor [38]. In a previous study, a breakfast with low GI and high GL gave better results in terms of cognitive improvement than a low-GI/low-GL breakfast [37]. This result indicates that the energy intake is as important as the origin, which in this study, mainly came from carbohydrates. Therefore, high- or low-GL diets differ by the amount of carbohydrate. However, low-GI/low-GL and low-GI/high-GL diets contain the same amount of total energy (twice that of low-GL diets). Furthermore, high GL induces a more important increase in the glucose level than high GI. Interestingly, the cortisol level was higher in the high-GI group, suggesting that low GI could protect against the stress response (cognitive tests in the study). Finally, the authors also reported that adolescents fed the high-GL meal felt more confident, less sluggish, and less hungry or thirsty before the tests. The low-GI fed group, on the other hand, were happier, more alert and less nervous and thirsty before the tests. Previous research supports the findings of Micha et al., showing improved alertness and decreased fatigue following a low-GI/high-GL breakfast [44]. However, even so, cognitive test results are not affected by GL, since the observed effects were similar in all groups. This observation confirms the results mentioned above indicating that a high glucose level is not the main vehicle of cognition amelioration. Moreover, the glucose increase measured could suggest stimulation of the hypothalamic–pituitary–adrenal axis. In turn, this activation would result in the increased cortisol level measured. This loop would then serve as an anticipatory response to a stress [37]. Consequently, this decreased stress will contribute to the improved results obtained during the cognitive tests. Interestingly, a low blood glucose level after a fast alters the hypothalamic–pituitary–adrenal axis [45]. Low GI could then be responsible for lowering the cortisol level by inducing a lower blood glucose level. During a learning and memory task, this effect could represent an advantage for both memorizing and recalling (Table 3).

According to studies in both adolescent and older populations, the glucose level and the source of carbohydrates are important in cognition, memory, and mood. However, healthy young populations display a strong effect, while the elderly only present a positive effect of a low-GI diet in groups with poor glucose regulation. It is possible that aging alters the sensitivity to GI/GL. Moreover, elderly studies have been conducted via questionnaires. This means that there could have been a large variety of meals, as well as nutritional habits, within the group studied. This could have influenced the results observed. Finally, it is worth noting that the elderly are cognitively healthy, but can display other medical histories and medications that could alter the real impact of diet on cognition. A longitudinal study should help determine how a specific dietary habit followed for a long time period will impact cognition in healthy populations. Additionally, the selection of healthy participants could help determine the effect on the development of age-related diseases, including cognitive deficits. To date, there is no study on the impact of a GI diet on cognition, mood, or memory in an adult population exhibiting healthy conditions. Furthermore, the role of the glucose supply in brain activity is supported by the demonstration of blood glucose level variation, depending on a mental task and emotion in younger adults (≈25 years) [46]. More recently, younger adults (18–23 years old) and older adults (65 to 85 years old) were tested for memory recognition after a glucose or placebo injection. Only the older group showed an improvement in cognition. However, as previously mentioned, this population displays poor glucose control [67]. Overall, it is suggested that the glucose supply is still critical for maintaining a normal brain function, as shown by a decreased blood glucose level during high brain activity [46,68]. In addition, a higher cognitive decline in people with poor blood glucose control or insulin resistance was previously observed [47] (Table 3).

It is worth noting that Philippou et al. failed to draw a conclusion on the consensual effect of GI on cognition in their review [33]. Nevertheless, in regard of the key role of the blood glucose level in cognition, GI values can still be important. In fact, in previous studies, the task or parameter analyzed (recognition, learning, memory, mood, accuracy, etc.) and the population studied (different ages, ethnicities, and health conditions) could have affected the results, making comparisons difficult. Finally, the meal composition and time of the experiment (morning vs. noon vs. evening) are also important parameters that change between studies. Despite all of these issues, the review by Philippou et al. helps us make assumptions about possible mechanisms by which GI affects cognition. First, it is suggested that the blood glucose concentration, rather than the amount provided, influences memory enhancement. Therefore, since high GI induces a transient increase in blood glucose, while a low GI leads to a more sustained increase, although lower, a low GI is more likely to induce long-term effects [43,69,70]. In support of that, it has been described that the GI enhancement of cognition appears in the post prandial phase following a meal [43,69,70]. Another putative mechanism involves insulin that is affected by GI and plays a role in cognition. Indeed, insulin resistance alters this role in cognition regulation [71], while low GI improves the insulin sensitivity and should thus improve cognition [72]. Cortisol is another hormone involved in cognitive function modulation via the above-mentioned hypothalamic–pituitary–adrenal axis stimulation induced by a lower blood glucose level due to low-GI food [37]. Consequently, low GI is associated with a decrease in the stress response, triggering improved results in cognitive tasks (Table 3).

Altogether, it is difficult to conclude a clear effect of GI on cognition. Nevertheless, the schoolchildren studies gave the most solid results. Indeed, the results are interesting since GI and nutritional approaches could be important in childhood and during learning processes. Therefore, efforts need to be made to better understand the impact of nutrition on cognition. This knowledge would be useful for setting up new nutritional recommendations for children. Finally, the studies presented so far do not address in depth the mechanisms involved in cognition and the role of GI/GL. However, pathological studies have allowed the effects of diet on brain function to be tested with more attention.

## 3. Low-GI Diet and Neurological Dysfunctions

The previous studies are somewhat confusing and difficult to interpret, in addition to providing very little insight on the mechanisms involved. Interestingly, brain dysfunction research generates more information on the nutritional impact on brain function. Indeed, nutrition therapies used in brain dysfunctions have presented promising results in improvement of the pathology. Therefore, several reports on epilepsy, seizure, ASD, AD, and others have studied brain function after diet interventions. It should be noted that these studies have also permitted hypotheses on the mechanisms involved to be drawn. Therefore, such research could provide greater insights on the mechanisms in play in neurological diseases and metabolic regulation.

### 3.1. Epilepsy

Epilepsy has been associated with a ketogenic diet (KD) intervention for a long time [5]. KD is a low-carbohydrate and high-fat diet. Additionally, because of its side effects (ketoacidosis, hyperlipidemia, and hypoglycemia), other diets have been tested more recently (Table 2). Most of these diets are modified KDs with more carbohydrates, including a low-GI diet [73]. Overall, these diets have been shown to decrease the number of seizures in pediatric patients by at least 50% [74] (Table 3). Several attempts to elucidate the mechanisms in play have led to different hypotheses involving ketone bodies, mitochondria, or gene regulation. In these diets, the decrease in carbohydrates is compensated for by a higher amount of fat, which induces a shift in nutrient utilization in favor of lipid oxidation. This high rate of lipid oxidation in turn generates ketone bodies [8,75]. Ketogenesis usually occurs in the liver during fasting periods, but also in type 1 diabetes (due to a defect of glucose utilization because of the absence of insulin) or obesity. Neurological disorders have been associated with decreased glucose utilization. In this condition, the brain becomes dependent on ketone bodies for energy supply [12]. Ketone bodies are used as an alternative source of acetyl-coA, which is paralleled by a consumption of oxaloacetate. This stimulates the Krebs cycle and increases α-ketoglutarate. In turn, α-ketoglutarate forms high amounts of glutamate by consuming aspartate, whose level is then lowered. Finally, the glutamate produced is decarboxylated by the glutamic acid decarboxylase to produce the inhibitory neurotransmitter GABA (γ-aminobutyric acid) [76]. Interestingly, GABA is described as an anti-seizure substance, and drug agonists targeting it are used in epilepsy [48,77,78,79,80]. Among these molecules, benzodiazepines enhance GABA’s action [81]. In support of this mechanism, children treated with low-carbohydrate diets (low GI) present high levels of GABA in their cerebrospinal fluid [82].

Another hypothesized mechanism of ketone bodies is that they directly enter mitochondria and the tricarboxylic acid cycle (TCA) to be oxidized. In turn, the stimulated oxidative metabolism inhibits phosphofructokinase 1 and glycolysis. This direct metabolization of ketone bodies decreases the ATP produced in glycolysis that will open the ATP sensitive potassium channels (K-ATP) and decrease neuronal activity [83]. In support of that, a genetic model of drosophila exhibiting seizure-like activity upon mechanical stimulation showed a reduced number of seizures when given ketone bodies. Moreover, blocking the K_ATP_ channels or adding a GABA antagonist has been shown to partially reverse the effect of ketone bodies [84]. This partial effect suggests that other mechanisms are also involved. For instance, other studies indicate a blockade of vesicular glutamate transporter (VGLUT) transfer to the synapse by ketone bodies. Such a blockade will decrease the excitatory glutamate neurotransmitters and thus neuronal activity [85,86].

Mitochondria have also been linked to the anti-seizure effect of KD. Here, ketone bodies increase mitochondrial respiration and NADH oxidation, inhibit reactive oxygen species (ROS) production, and enhance ATP production [87]. All of these should prevent the mitochondrial permeability transition (mPT), which ultimately leads to cell death [88]. In support of that, mice with recurrent epileptic seizures show an increased threshold of mPT. This anti-mPT effect depends on cyclophilin D modulation (part of the mPT). Furthermore, learning and memory and long-term potentiation are decreased in these mice, suggesting a role in cognition [89]. However, this theory is contradictory to previous activity decreasing ATP production. Therefore, more research is needed to completely determine the role of ketone bodies in ATP production. Nevertheless, mitochondrial activity has been linked to neuronal function and diseases, while KD is known to improve mitochondrial activity [90]. Therefore, KD induces decreased mitochondrial ROS production compared to a normal chow, via changes in the gene expression of the oxidative pathway. Another possibility is that the increased NAD/NADH ratio induced by ketone bodies dampens ROS production [91]. Ketone bodies also improve the consumption of O2 within the respiratory chain. By doing so, KD decreases the rate of ROS production by the mitochondria. Since seizure is associated with oxidative stress, KD or modified KD could diminish the seizure occurrence in epileptic patients [92]. Moreover, besides having a direct effect on ROS production, KD also stimulates the antioxidant protein catalase, whose expression is stimulated by the activated peroxisome proliferator activated receptor γ2 (PPARγ2) transcription factor [92]. Here, PPAR activation occurs after histone hyper-acetylation following the inhibition of histone deacetylases (epigenetic regulation) [93,94]. In turn, PPAR upregulates antioxidant genes and downregulates pro-inflammatory genes (NFKappaB, cyclooxygenase 2, and iNOS) [95]. Histone deacetylase inhibitors are used as anti-inflammatory and anti-epileptogenic molecules [96]. Interestingly, ketone bodies are shown to inhibit histone deacetylase, although the precise mechanism still needs to be determined [97].

Finally, Rahman et al. hypothesized that ketone body neuroprotection could rely on the G-protein coupled receptor GPR109 recently identified, which is activated by ketone bodies and found in the brain. Moreover, mice fed a KD or infused with ketone bodies showed a decrease of ischemic infarcts dependent on GPR109. Furthermore, the authors demonstrated that the activation of GPR109 by ketone bodies occurs in infiltrated monocytes and macrophages. In turn, these cells produce prostaglandins and induce an anti-inflammatory response that reduces seizures [98,99]. Finally, the anti-inflammatory role of ketone bodies inhibits NLRP3 inflammasome assembly through the blockade of K+ efflux. However, the precise mechanism remains to be elucidated [100].

### 3.2. Stroke

Vegetarian diets (low GI and GL) have been described to lower the risk of stroke [101,102] (Table 3). Therefore, a relationship between GI/GL and stroke likely exists. On the other hand, a high-fructose diet that should have a high GI worsened post ischemic brain injury in rats [103]. In this study, the authors highlighted an important effect in the hippocampus with increased inflammation while neuronal plasticity was decreased, in parallel with neuronal loss. These changes lowered the neuronal performances during ischemic recovery compared to normal chow fed mice. These results support a role of the diet during the recovery period following a stroke. Here, the glucose level is also at play during the acute stage of a stroke and the recovery period. Therefore, diabetes is a risk factor for stroke [49,50]. The oxidative stress associated with hyperglycemia is expected to be the mechanism involved in the poor outcome following a stroke. This suggests that better glucose level control could help decrease the risk of stroke [104]. Diet intervention studies have provided interesting results in this regard. Therefore, a high-GL diet is associated with a poor outcome in patients with acute ischemic stroke [105]. Recently, Song et al. found that, in diabetic patients, this poor outcome does not depend on diabetes, but only on the diet’s effect on glycemic variation. Furthermore, the authors suggested that chronic hyperglycemia is the main anticipated cause. Chronic hyperglycemia induced by a high-GL diet was previously shown to induce a poor outcome after a stroke [106,107]. One possible explanation for this is the cerebral hypo-perfusion or edema associated with hyperglycemia, which leads to poor recovery from stroke [51,52]. Moreover, stroke is often linked to cytotoxicity, whose association with hyperglycemia dampens the recovery [53]. In addition, mitochondrial dysfunction due to lactate production from glucose metabolism has also been suggested. Here, lactate acidifies the intracellular environment and triggers mitochondrial dysfunction [108]. A rapid increase in blood glucose also leads to oxidative stress and endothelial dysfunction observed in diabetes [109]. Furthermore, high increases followed by periods of low levels are more deleterious than a continuous rise. Interestingly, high GI/GL increases glucose transiently, and thus has a negative effect on stroke outcome. Indeed, a dysfunctional endothelium has been described as a negative factor for acute ischemic stroke outcome [110]. Moreover, high-GI/GL diets induce insulin resistance [31] that will increase serum levels of fibrinogen and the von Willebrand factor (endothelial function), thus increasing the risk of stroke [111,112]. Therefore, high GI/GL should induce hypercoagulability and an increased risk of thrombosis. This phenotype is paralleled by an increase in small vessel diseases that could impair stroke outcome [113]. Finally, in a cohort of Chinese women followed for 10 years, a positive association between GI and the risk of stroke was observed, supporting the previous hypothesis [114]. Furthermore, the low-GI Mediterranean diet is associated with a reduction in the risk of stroke [115]. Finally, Lim et al. studied the role of diets in stroke recovery and cognition impairments. In their study, the authors described that glycemic variability with hyperglycemic episodes is detrimental to cognitive function recovery. Therefore, a diet avoiding amplitude oscillations for glycemia is more suitable for both vascular and cognitive function recovery after a stroke [116].

Overall, evidence indicates that high glucose increases induced by diet impact both the risk and outcome of stroke. The mechanisms in play are related to mitochondria and inflammation, as well as hypercoagulation. Moreover, chronic and rapid hyperglycemia following a meal is deleterious for both the risk and outcome. Therefore, a low-GI diet that induces a sustained elevation in blood glucose should be beneficial for recovery after a stroke, and decreases the risk of occurrence by improving cardiovascular function.

### 3.3. Alzheimer’s Disease (AD)

Diets are widely use in AD to help delay or slow the development of the disease [54,55]. Additionally, the relationship between AD and metabolic disorders (insulin resistance) means that this disease is considered as type III diabetes [117]. Therefore, nutritional approaches to studying AD have provided insights into the brain function in the pathology related to diet changes. Recently, it was reported that a high-GI diet increases the accumulation of amyloid β (Aβ) in brains of the elderly, which is a marker of AD, as well as a risk factor for the onset of the disease. In addition, these individuals showed a cognitive decline, and PET scan imaging demonstrated an increased association between Aβ accumulation and a high-GI diet [118] (Table 3). This interaction being independent of all other factors (age, sex, and education) indicates that high GI is highly involved in Aβ accumulation in aging populations and represents an increased risk factor for AD. In support of a key role of diet in AD onset, high-fat diet (HFD)-induced insulin resistance increases brain Aβ accumulation. However, mice deleted for IRS2 (insulin signaling) become insulin resistant, but do not accumulate Aβ, unless fed an HFD. Others have also shown that Aβ accumulation is accompanied by pTau aggregation (characteristic of AD) in an insulin-resistant AD mice model. Finally, oxidative stress and inflammation have been described in this mice model [119].

Overall, these results indicate that the Aβ burden is related to the diet, confirming that nutritional adjustments could help prevent AD onset and slow its progression [120]. In support of this, a recent review describes that a healthy diet can decrease the risk of AD by lowering oxidative stress and dampening Aβ accumulation [56]. Moreover, this review discusses the anti-inflammatory effect of a healthy diet. In AD, inflammation is caused by Aβ accumulation that stimulates the recruitment of microglia and astrocytes parallel to interferon gamma (IFNγ), interleukin 1β (IL1β), and tumor necrosis factor α (TNF α) secretion [57,121].

AD is characterized by a decreased glucose uptake and utilization by brain cells. However, the brains of AD individuals can still use ketone bodies [58,122]. Therefore, KD could be used in AD to slow progress of the disease or to delay the cognitive deficit. A medium-chain triglyceride diet (MCT diet) is a diet less restrictive in carbohydrates, but still ketogenic, and is used in AD [58]. The low content of carbohydrates forces the organism to use lipid oxidation as the energy source, which also produces ketone bodies. Therefore, such diets have a low impact on glycemia, making them low-GI diets. Brain energy metabolism and glucose uptake decreases are accompanied by mitochondrial dysfunction in AD [58]. Brain cells increased the utilization of ketone bodies during brain energetic deficiency, leading to a new “neuroketotherapeutic” strategy to compensate for the lack of glucose as an energy source [58]. Besides this energetic role, ketone bodies are also involved in neurotransmission and a reduction in oxidative stress and inflammation relevant to AD. Ketone bodies involve the mitochondria and their functional changes. As AD is associated with mitochondrial dysfunctions [123], ketone bodies could thus improve the disease phenotype. KD has also been shown to increase the number of mitochondria in the hippocampus, which could contribute to the improvement of AD [124]. Furthermore, since ketone bodies produce fewer ROS than glucose, they can participate in a decrease in the oxidative stress in AD [59,125]. This decrease in ROS production could be induced by stimulating the expression of uncoupling proteins (UCP), as shown previously [126,127]. Another possible mechanism is that reducing glutamate transport and improving GABA activity decreases the excitability of neurons and thus ROS production (see Section 3.1). In addition, KD was shown to upregulate antioxidant proteins (MnSOD, Glutathione, and Nrf2) [58]. Finally, KD inhibition of histone deacetylase can allow the expression of proteins improving cellular homeostasis and function (brain-derived neurotrophic factor (BDNF)), and in turn cognitive deficit in AD patients [128,129,130,131,132]. In support of the benefit of KD diets, mice models of AD also show decreased Aβ accumulation in the brain when fed a KD [58], while in humans, a medium-chain triglyceride (MCT) diet has been shown to result in an encouraging improved memory or at least the stabilization of cognitive function in AD individuals [58]. The direct effect of a KD in humans has been summarized by Taylor et al., who present an improvement of almost all the memory and verbal communication tests in AD patients under a KD [58] (Table 3).

Altogether, these results support an improvement of AD pathology by decreasing the carbohydrate supply and thus lowering the GI of the meals. The benefits depend on ketone bodies that help maintain neuronal activity, decrease oxidative stress, and stimulate gene regulation. However, a KD displays important side effects. Therefore, more research should be conducted to help better understand the mechanisms at play, and eventually develop a therapeutic approach targeting these mechanisms. Moreover, other low-GI diets could have similar beneficial effects to KDs, and thus must be tested. Overall, although there is solid evidence for ketone body involvement, the diets used in AD have several other effects that could be involved. Therefore, conducting more research to understand the role of low GI on the AD phenotype could help develop a diet adapted to the disease.

For instance, the Mediterranean diet (MD) is also protective against cognitive decline in AD [55]. MD is characterized by: a high intake of vegetables, legumes, fruits and cereals, and extra virgin oil (unsaturated fatty acids); low intake of saturated fats and meat; a moderate intake of fish; and a low to moderate intake of dairy products and wine during meals [133,134]. MD is low in carbohydrates, while high in fibers, and so can be classified as a low-GI diet (Table 2). MD is known to have numerous health benefits in cardiovascular or metabolic diseases, and also in cognition (lower decline) and in reducing the risk of dementia or AD [135,136]. Furthermore, people with mild cognitive impairment fed an MD show a decreased risk of AD onset, and improved memory, delayed recall, and global cognitive function [133]. Different studies suggest antioxidant, anti-inflammatory, or cognitive function enhancement, depending on the nutrient components. For instance, olive oil, as a main source of fats enriched in omega-3 and phenolic acid, is considered to be a main factor [133]. Olive oil decreases the glycemic response to a high-GI meal [137]. As such, olive oil helps lower the GI of a meal, and thus decreases the glycemic increase induced by a meal that could participate in the effects observed. In addition, since the carbohydrate content of MD is low or non-digestible, ketogenesis is expected and repeats the action described above. Accordingly, a modified Mediterranean–ketogenic diet has been described to be associated with changes in AD biomarkers in cerebro spinal fluid (CSF), suggesting that ketone bodies could be involved [138]. In addition, the observed changes in the brain could result from gut microbiota alterations, suggesting that nutrient digestion and/or absorption are important steps in AD onset and cognition. These results reinforce the relationship between nutrition, metabolism, and AD and cognitive function. Microbiota have been shown to interact with brain function through the metabolism of dietary fibers in certain bacteria that produce propionate or butyrate (short chain fatty acids (SCFA)). In turn, these SCFA exert brain effects via histone deacetylase, transcription factors, or antioxidant regulations. All of these effects will then provide neuroprotection and therefore protect against neurodegenerative disorders [139]. On the other hand, Western diets containing processed food and carbohydrates decrease the number of bacteria producing SCFA. By doing so, these diets have deleterious effects on brain and cognition. Interestingly, highly processed food will most likely have a high GI, while dietary fibers are low-GI carbohydrates, indicating that low-GI foods are protective against neurodegenerative diseases.

The gut–brain axis and nutrition have also been shown to participate in the pathophysiology of Autism Spectrum Disorder (ASD) [140]. Moreover, Parkinson’s disease (PD) is improved by a KD. Altogether, nutrition and GI could have an impact on other neurological conditions.

### 3.4. Others: Dementia, Depression, Mental Health, etc.

Metabolism is often associated with pathological brain conditions. Therefore, the AD and PD risk increases with malnutrition and insulin resistance, while diet control is protective (138). Furthermore, insulin has been shown to increase dopamine transporter mRNA levels in the substantia nigra [141]. Therefore, it is assumed that a high-GI/GL diet could prevent PD by inducing high insulin secretion [142] (Table 3). However, the study suggesting a role for insulin is controversial, since high GI induces insulin resistance, while low GI improves insulin sensitivity [31]. Nonetheless, whilst the population studied presented a lower rate of PD while consuming high-GI food such as rice, it is likely that other dietary factors could be involved. Indeed, the Japanese diet is considered a healthy diet contributing to the expanded life expectancy observed in Japan. Therefore, cardiovascular-related death and neurodegenerative disease rates are amongst the lowest in the world [143]. The Japanese diet is characterized by raw ingredients used in meal preparation. The diet is low in fat and calories because most of the foods used are vegetables, fish, meat, and rice, providing an excellent nutritional balance. This diet is low in red and processed meats, whole milk, refined grains, sweet drinks or alcohol, candy, and sweets, but enriched in fruits, vegetables, stevia sweeteners, and whole grains and fish products (Table 2). Overall, the Japanese diet is expected to be low GI (stevia has a GI/GL = 0) [142]. Therefore, the observed decrease in PD in the study of Murakami et al. could be due to an improved insulin sensitivity, rather than increased insulin levels. It was also described that a high-fat (high-GI) diet is responsible for a decreased number of dopaminergic neurons in the substantia nigra due to a reduced PPAR and inflammation. Therefore, a low-fat diet such as the Japanese diet could prevent these PPAR and dopaminergic neuron decreases and protect against PD [144]. The MD is also associated with a decreased risk of PD. Here, the microbiota changes induced by the diet are interesting since they are related to dietary fibers (lowering the GI) as a carbohydrate source in the diet. In addition, it strengthens the importance of the gut–brain axis in brain function and disease. Dietary fibers are known to stimulate the production of SCFA that could be part of the mechanism of protection against PD in the MD. Indeed, SCFA can improve insulin sensitivity, reduce inflammation, and stimulate brain-derived neurotrophic factor (BDNF) production, helping to protect against PD. Another possible mechanism occurs through the antioxidant-rich content of the MD. It should be noted that some of the antioxidant products in the diet can also stimulate SCFA production, reinforcing the role of these molecules in brain function and pathology [145]. In support of that, the SCFA concentration in the feces of PD patients is decreased compared to control individuals [146]. However, Shin et al. also observed an increase of plasma SCFA correlated with the PD severity [147]. This result suggests putative SCFA leakage from the intestinal lumen into the bloodstream. Nevertheless, further studies are needed to clearly address the impact of SCFA on PD, and more generally, the impact of diets.

Depression and anxiety are other brain diseases that exhibit a relationship with energy homeostasis [60,61]. Furthermore, depression has been studied in relation to GI/GL [148]. Although the results are inconsistent, it seems that high-GI/GL diets increase the risk of depression, as well as aggravate the score of the disease [148] (Table 3). Rodent studies showed that HFD impaired 5-HT neurotransmission, which increases anxiety behavior. The same group also reported decreased anxiety following Connexin 43 downregulation or phosphorylation, or treatment with metformin (activator of AMPK) [149,150,151]. In this context, metformin triggers a decrease in circulating branch chain amino acids (BCAA) that in turn contribute to the anxiolytic and antidepressant effect. BCAA have been previously linked to glutamate transport between intracellular to extracellular medium [152]. It was also observed that a metformin decrease of BCAA is due to the inhibition of ketone-derived BCAA production [153], suggesting that ketone bodies could be involved in depression and anxiety. Interestingly, in addition to ketones, anxiety and depression have been associated with oxidative stress, inflammation, and gut–brain communication [66,154]. Moreover, drugs have been developed to target glutamate signaling and its recycling in the synapse [155]. Therefore, it is likely that diet interventions could involve similar processes to those described above.

Autism Spectrum Disorder (ASD) is a neurological condition associated with behavioral and social interaction defects and that shows energy homeostasis dysregulation [156]. In fact, type 2 diabetes and obesity during pregnancy are risk factors for ASD in offspring [156,157]. Chronic inflammation observed in metabolic disorders and immune system activation that occurs during pregnancy appear to be key in the risk of ASD [158,159,160]. It is noteworthy that chronic inflammation can be decreased by a low-GI diet in obese subjects [161]. On the other hand, a high-GI diet increases advanced glycation end products (AGEs) that are involved in inflammation during obesity and diabetes. Indeed, the AGE activation of specific receptors results in C Reactive Protein (CRP) production and oxidative stress [162,163]. In support of this possible mechanism, Currais et al. demonstrated that mice offspring from high-GI-fed parents displayed increased brain inflammation, reduced neurogenesis, and characteristic ASD behaviors [164]. Dietary strategies have triggered improvement of the ASD phenotype after birth. For instance, KD given to a mice model of ASD improved the social behavior and decreased repetitive behaviors [165]. In another attempt, gluten-free foods decreased the inflammatory grade and improved the ASD phenotype. However, only some of the subjects showed improvement, while others failed to observe a benefit [166,167]. These studies, which used different diets, suggest that a specific nutrient or compound could be at play. Furthermore, inflammation is involved in the onset of the disease; however, other brain energetic alterations could also be present, but remain to be determined. Therefore, more research on both the understanding of ASD brain alterations and diet interventions to determine the role of specific nutrients or metabolic products needs to be conducted.

Microbiota are also associated with ASD behaviors [62,140,168]. Even if the role of gut microbiota is poorly understood, amino acid metabolism and inflammation are possible mechanisms participating in the phenotype observed. A low-GI diet induces changes in microbiota that are associated with ASD improvement. Therefore, the participation of a product with low-GI carbohydrates could be important. Finally, the ASD mice model shows decreased blood levels of methionine, also described in humans [63,64,65]. Interestingly, a high-GI diet also decreases the methionine levels [164]. Methionine is a precursor for DNA methylation and gene regulation [169]. In comparison, a low-GI diet maintains higher levels of methionine, suggesting that diet could improve ASD through epigenetic regulations. In line with the role of microbiota and low GI, sulforaphane, produced from low-GI vegetables (cauliflower and broccoli), has been identified as a putative treatment in ASD. Indeed, sulforaphane given to autistic children improved their behavioral phenotype. Moreover, although the mechanism of action is not known, sulforaphane has been described to modulate oxidative stress, methylation, or apoptosis, and to be a potent neuroprotective molecule [170,171,172,173,174]. Overall, the identification of this molecule supports a beneficial role of a low-GI diet in ASD. It is noteworthy that, similar to other neurological conditions, ASD improvement through the diet is linked to oxidative stress, gene regulation, and inflammation. Moreover, a sulforaphane extract from vegetables is a promising molecule for the treatment of ASD. Further, other molecules produced or present in low-GI foods could also exist and participate in the beneficial effects, and would thus be useful in other neurological diseases (Table 3).

Intriguingly, oxidative stress, inflammation, ketone bodies, the gut–brain axis, microbiota, glutamate metabolism, and neurogenesis are all involved in the neurological conditions described. All of them can also be affected by the diet and especially the carbohydrate content and source. Therefore, low-GI diets are likely to improve neurological disorders, but may also be of interest in physiological conditions, since they help improve cognition and neuronal activity. A more generalized use of a low-GI/GL diet could help prevent or delay the onset of neurological disorders, while it could also help during the growing up period and in learning performances in childhood. 

Although most of the mechanisms described appear to be common to the diseases described and are linked to diet intervention changes, no exact mechanisms and molecules involved in these beneficial effects have been precisely identified. Nevertheless, a lot of common pathways indicate a role of ketone bodies and SCFA. Therefore, more studies to determine diet involvement in the production of these molecules should be conducted to determine precise diet recommendations. Considering this, neurological disorders are likely to be successfully targeted by nutritional therapies.

## 4. Glycemic Index and Brain Regulation of Energy Homeostasis

GI/GL values predict the impact of food on the metabolic response, such as blood glucose and insulin levels. Therefore, a high GI/GL will induce a rapid and high increase in blood glucose and insulin, while a low GI/GL will induce a slow and limited increase of blood glucose that slightly affects insulin levels. Due to these effects, diets based on low-GI foods have been developed to help control weight and prevent cardiometabolic disorders [21,175]. However, studies to address GI/GL’s effect on weight loss or glucose management in diabetic people have given rise to contradictory results. Among other reasons, the differences can likely be attributed to a range in GI values for the same food making food choices based on such criteria more difficult. In addition, GI only considers the carbohydrate content of food, while lipids, proteins, and vitamins can also influence the metabolic impact of the food [176].

The brain consumes a large part of glucose from the body for its activity and needs a constant supply [12]. Therefore, according to the “selfish brain” theory, the brain competes with the body for glucose. Because of this, the brain sends a message to increase glucose availability when its activity increases. Furthermore, the brain is overly sensitive to glucose availability, and responsive to changes in the glucose supply [13]. Such sensitivity makes the brain a key regulator, not only for its needs, but also for maintaining a constant glucose level in the blood [15]. Blood and brain parenchyma glucose levels are linked through transport across the blood–brain barrier via GLUT1 [177]. While hyperglycemia decreases GLUT1 expression, a drop in blood glucose will stimulate GLUT1 expression [178]. However, the brain glucose level does not change in association with the blood concentration [179]. Therefore, micro-dialysis experiments have revealed a brain to blood glucose ratio of about 0.5. Furthermore, this ratio decreases when blood glucose increases [180]. This observation could be the result of a decreased GLUT1 expression that limits glucose uptake by the brain. Altogether, these results indicate a tightly regulated glucose supply to the brain to prevent both hypo- and hyperglycemia from reaching the brain.

Physiological conditions involve complex inter-organ communication to keep body weight and energy homeostasis in a balanced state. The brain plays a crucial role by integrating information on the body’s energy status and adapting food intake and energy expenditure via signals sent to peripheral organs [181]. Furthermore, specific areas of the brain are involved in the sensing of glucose, as well as hormone concentration changes (insulin, ghrelin, leptin, etc.) [182]. In this regard, GI/GL is expected to act in the brain control of energy homeostasis. However, little is known about the impact of high or low GI/GL on those mechanisms. Nevertheless, due to changes in blood insulin and glucose levels, changes in brain responses are likely to occur. Whether these changes are due to direct (glucose or insulin level) or indirect (hormonal, nutrient, or cytokine level changes due to the meal) effects on the brain control of metabolism should be determined. Moreover, the relationship between GI and metabolic disorders remains unclear, despite several studies having been conducted [183,184,185,186,187]. Intriguingly, the mechanisms involved in cognition or neurological disorders have been described in brain energy homeostasis regulation. Therefore, ROS signaling, mitochondrial activity, the gut–brain axis and SCFA, ketone bodies, astrocyte–neuron communication, glutamate metabolism, and gene regulation and neurogenesis or plasticity have been involved in brain metabolic regulations.

Nevertheless, so far, only a few studies have tried to determine the impact of GI/GL on the brain control of metabolism. However, nutrition can affect the body weight, insulin sensitivity, adiposity, and other metabolic parameters. Therefore, we will discuss results suggesting a GI/GL impact on the brain control of energy homeostasis.

### GI/GL and Brain Glucose Detection

Within the brain, specific regions are dedicated to the sensing of signals involved in energy homeostasis regulation. The hypothalamus is the first area to sense these peripheral signals on the energy status of the body. Within the different nuclei forming the hypothalamus, various neuronal populations have been well described [14]. Furthermore, glucose sensing and the regulatory response to the periphery have been well characterized in the hypothalamus [15]. This precise sensing of blood glucose is possible due to the blood–brain barrier permeability being weaker in this area of the brain. Due to this, the impact of foods on the blood glucose level is directly integrated by the hypothalamus to restore euglycemia. In this regard, GI/GL should involve a hypothalamic activation of neuronal circuits of glucose and energy homeostasis regulations. Low-GI food leads to a controlled increase in the blood glucose level that is progressively returned to euglycemia. In this case, insulin release is only limited, and the glucose level is decreased due to tissue utilization. On the other hand, high GI/GL results in a high increase of glucose levels in the first phase. Such a rise in blood glucose stimulates brain hyperglycemia detection. In turn, high glucose excited (HGE) neurons are activated, leading to the release of neurotransmitters (GABA in VMN and NTS or anorexigenic neuropeptides POMC/CART in the ARC) [188]. In a second phase, this should lead to a decreased blood glucose level that involves the same area, but stimulates other neurons called glucose-inhibited neurons. The activation of these neurons occurs when glucose levels decrease. Following their stimulation, the brain will send adaptive signals to stimulate food intake and glucose production [189]. The brain sensing a high glucose level will induce a peripheral response through inducing insulin release by the pancreas [190]. This response contributes to the rapid decrease of the glucose level and the hypoglycemia induced. Following this second phase, hypoglycemia detection is activated and involves the brain in contributing to stimulating the food intake by activating orexigenic neurons.

Interestingly, the redox balance is involved in both hyper- and hypoglycemia sensing. Therefore, hyperglycemia induces mitochondrial ROS production in the hypothalamus. The inhibition of this ROS production inhibits the insulin secretion normally observed due to a lack of nervous activation [191]. In addition, changes in the morphology of mitochondria are required to finely regulate this ROS signaling [192]. During obesity and diabetes, a hypersensitivity to glucose is observed. This hypersensitivity is characterized by insulin secretion by the pancreas after detection of a glucose level lower than the usual level. This hypersensitivity is linked to dysfunctions of mitochondrial respiration activity. Consequently, ROS levels are higher than in the non-obese group. Finally, an antioxidant treatment to decrease the ROS level in the hypothalamus restores the normal glucose sensing function, demonstrating the importance of redox balance [193,194]. Dysfunctions of mitochondrial dynamics and ROS signaling have also been shown during metabolic unbalance [195]. Hypoglycemia detection also depends on ROS signaling. Here, ROS are produced during normal hypoglycemic counter-regulation. However, after recurrent hypoglycemic episodes, there is no ROS production [196]. These results support a key role of mitochondria and the redox balance in the hypothalamic regulation of energy homeostasis, as also shown in other studies [197,198,199,200,201]. In addition, lipids, insulin, and ghrelin signaling in the hypothalamus also participate in ROS signaling. Therefore, according to the mechanisms putatively involved in a low- vs. high-GI diet, and its impact on ROS production (see Section 3), it is likely that the hypothalamic regulation of metabolism will be affected by the GI of a food or diet. For instance, a high-GI diet could mimic a recurrent increase in ROS and therefore disrupt the mechanism of hypoglycemia counter-regulation. Additionally, recurrent increases in glucose and insulin could participate in the development of insulin resistance in the brain, as has been observed for a high-GI diet in peripheral tissues [31]. On the other hand, a low-GI diet would produce ketone bodies that are inhibitors of ROS production by interfering with the mitochondrial respiration, as described above [90,91,92,202,203,204,205,206,207,208]. Interestingly, ketone bodies have recently been shown to be involved in metabolic disorders and the brain control of energy homeostasis, notably by affecting the food intake [202,203,204,205,206,207,208]. Discrepancies in results could be due to various parameters, including the origin of ketone bodies. Indeed, a high-fat diet is associated with hyperketonemia induced by an increase in fat metabolism from the diet, while fasting ketone bodies are produced from lipid stores in the body. Therefore, the results observed can be altered by other signals, such as fatty acids, hyperglycemia, hyperinsulinemia, and leptin, among others, during the consumption of a high-fat diet. A high-GI diet probably falls into this situation since it is high in carbohydrate and high-GI foods. A low-GI diet, however, will induce ketone body production from the fatty acids produced from adipose tissue. Moreover, in addition to ketone bodies produced in the liver, in this condition, astrocyte ketogenesis will also be stimulated [209,210]. Indeed, it is possible that the signal exhibited by ketone bodies produced by astrocytes locally would be different from that displayed by hepatic ketone bodies that would affect the whole body.

Ketone bodies could also be involved in metabolism through the acetylation or methylation of genes, thus modulating their transcription, as described before [93,94,95]. Indeed, such gene regulation has previously been linked to the brain control of energy homeostasis [211,212,213,214]. Of particular interest, these processes have been shown to be involved in neurogenesis, brain plasticity, and neuronal function. Therefore, low-GI diet-produced ketone bodies should lead to changes in these mechanisms and modify the neural regulation of metabolism. Since ketone bodies are associated with fat metabolism, they would rather signal a lack of energy that would stimulate food intake or increase the glucose supply. However, this assumption remains to be tested, since others have described an inhibition of food intake by ketone bodies. Nevertheless, epigenetic mechanisms stimulated by low GI should help control the energetic balance, while high GI should lower ketone body production and thus block their signal. However, further studies are needed to completely understand the role of ketone bodies in the brain regulation of energy homeostasis.

Interestingly, ketone bodies are involved in changes in ATP production. Such a role has been described in neurological disorders. Either ketone bodies, by inhibiting glycolysis, decrease the ATP production, or by forming substrates for the mitochondrial respiration, maintain ATP production. Such hypotheses remain to be tested. Nevertheless, ATP levels would be responsible for the regulation of K_ATP_ channel closure [83,84,85,86]. Such a role of ATP production would then regulate the depolarization of glucose excited (GE) neurons that are K_ATP_-dependent channels [215].

Astrocyte ketogenesis also supports a role for glial cells in energy homeostasis. First, astrocytes provide energy to neurons to maintain normal activity. Furthermore, astrocytes play a role as a sensor of the metabolic status, and signal this information to the neurons [216,217]. Therefore, astrocyte–neuron communication is key in the regulation of energetic signal transport and sensing by neurons. Indeed, studies have described the role of astrocytes in the detection of leptin, glucose, fatty acids, and amino acids and their involvement in the metabolic balance [218,219,220,221,222,223]. The role of astrocytes in glutamate recycling is also associated with the lactate supply to neurons for neurotransmitter action [222]. In addition, disrupting the communication between astrocytes by blocking the Connexin 43 protein that participates in the astroglial network in the hypothalamus inhibits the glucose sensing response. This result supports indispensable transport through astrocytes for normal glucose detection [224]. The astrocyte-neuron lactate shuttle theory (ANLS) hypothesizes that astrocytes metabolize glucose taken up from the bloodstream, and then convert it into lactate in glycolysis. The lactate produced is then transferred to neurons, where it can be oxidized. Therefore, lactate serves as a signaling molecule for the blood glucose level. Accordingly, lactate infusion to the brain mimics the response obtained with glucose [225]. Overall, glucose changes associated with ANLS and glutamate recycling represent targets for a GI diet producing ketone bodies following similar mechanisms described in neuronal disorders. However, such a hypothesis has not been tested in the context of brain nutrient sensing.

Finally, the gut–brain axis is also involved in the brain regulation of energy homeostasis. In addition, the diet composition and GI alter the gut function via microbiota that have an effect on neurological disorders [226,227,228]. Here, the same molecules identified in neurological conditions are also known to affect brain circuits of metabolic control. Indeed, dietary fibers are expected to provide resistance to the onset of metabolic disorders, as well as the discussed effect on neurological disorders and cognition. Dietary fibers are found in low-GI food such as vegetables [16]. Therefore, the action of GI on microbiota will affect the brain control of energy homeostasis via SCFA. Here, SCFA have been described to have a satiating effect [37,229]. More recently, microbiota changes have been shown to activate POMC neurons (anorexigenic), reinforcing the role of the gut–brain axis in food intake control [230]. Other works indicate that leptin sensitivity inhibition and pro-glucagon and BDNF decreases induced by microbiota changes could participate in obesity [231]. Interestingly, leptin sensitivity and BDNF are linked to AD, strongly suggesting that microbiota could be key elements in the relationship between brain disorders and metabolism. Therefore, meal composition and the GI of foods and meals represent an interesting target in the elucidation of this relationship, but also in preventing, delaying, and treating the diseases.

## 5. Conclusions and Perspectives

**Figure 2:**
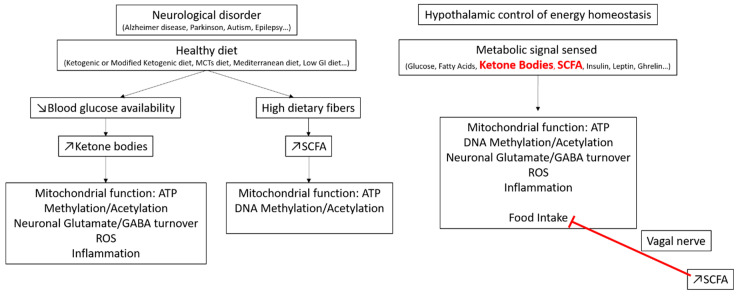
Schematic representation of the different mechanisms putatively involved in the beneficial effect of a low-GI diet on neurological disorders (**left panel**), paralleled with known mechanisms involved in the brain control of energy homeostasis (**right panel**).

GI/GL values have given rise to new diets to help people with metabolic diseases control their body weight and glucose homeostasis. Encouraging results on weight management and insulin sensitivity improvement have been observed. In addition, more historical diets, such as ketogenic or modified ketogenic diets, the Mediterranean diet, and the Japanese diet, which are likely to have a low GI, are associated with a healthy lifestyle. In addition to their metabolic improvement properties, these diets also exhibit positive effects on neuronal diseases. Therefore, people following these types of diets present a decreased risk of developing brain diseases while aging. Furthermore, these diets have also been used for decades to treat epilepsy, for example. However, even though it is known that these nutritional interventions improve wellbeing, little is known on the mechanisms involved. Interestingly, despite the primary role in metabolic control, the most important progress in the understanding of the brain impact of a low-GI diet has come from neurological condition studies. Nevertheless, it is worth noting that the mechanisms identified or hypothesized from these studies have been described in the context of the brain control of energy homeostasis. Among these mechanisms, the redox balance, mitochondrial function, ATP production, insulin sensitivity, gene regulation, astrocyte-neuron lactate shuttle, and neurotransmitter regulation are all involved (Figure 2).

Therefore, these mechanisms appear to be key in brain function, and an alteration of them could lead to neurological dysfunctions. These dysfunctions would then lead to neurological disorders or defects in brain regulatory mechanisms such as energy homeostasis. Therefore, the inability of the brain to precisely modulate energy needs for its own needs will trigger neurological dysfunctions.

However, a better understanding of these mechanisms is still needed to completely understand both neurological disorders and metabolic disorders. Moreover, improved knowledge could help to decipher the increasingly evident relationship between neuronal disorders and the metabolic balance.

Finally, a better understanding of the mechanisms involved in diets could help develop and improve nutritional recommendations for improving the health of people with these diseases. In addition, the diet could be a good target for preventing the development of both metabolic and neuronal disorders. Moreover, deciphering how these mechanisms involve diets could result from such studies and thus also improve cognitive function in healthy people.

## Figures and Tables

**Table 1:** Example of common foods with their corresponding serving size in g, glycemic index (GI) carbohydrates per serving size in g, and the resulting glucose load (GL).

Food	Serving Size (g)	GI	Carbohydrates Per Serving (g)	GL	Food	Serving Size (g)	GI	Carbohydrates Per Serving (g)	GL	Food	Serving Size (g)	GI	Carbohydrates Per Serving (g)	GL
Tuna	100	0	0	0	Fructose	10	23	10	2	Wheat	200	45	137	62
Salmon	100	0	0	0	Blackberry	60	25	4	2	Carrot Juice	250	45	24	11
Sardine	100	0	0	0	Grapefruit	120	25	11	3	Pineapple Juice	250	46	33	15
Mackerel	100	0	0	0	Milk, full fat	250	27	12	3	Banana	120	47	24	11
Crab	85	0	0	0	American Cheese	28	27	2	<1	Lasagna	125	47	19	9
Eggs (chicken)	50	0	1	0	Cottage	28	27	6	2	Penne	125	47	94	44
Beef	100	0	0	0	Chickpeas, boiled	150	28	30	8	Butter	5	50	0	0
Chicken	140	0	6	0	Lentil	200	28	40	11	Mayonnaise	15	50	0	0
Goat	30	0	0	0	Beans, kidney	150	28	25	7	Mango	120	51	15	8
Pork	85	0	0	0	Garlic	3	30	1	<1	Tortilla	50	52	24	12
Lamb	85	0	1	0	Vanilla extract	4	30	3	0	Blueberry	150	53	18	7
Ham	85	0	0	0	Buttermilk	245	31	12	4	Kiwi fruit	150	53	16	9
Turkey	85	0	0	0	Lime	67	32	7	<1	Date	60	54	33	21
Duck	140	0	0	0	Broccoli	80	32	4	1	Orange juice	250	55	26	14
Rabbit	85	0	0	0	Artichoke	150	32	14	4	Corn, Sweet	150	55	32	18
Macadamia	28	10	4	<1	Cauliflower	100	32	5	2	Cranberry Juice	250	55	33	18
Pecan	28	10	4	<1	Green Bean	55	32	4	1	Honey	25	55	20	11
Almond	28	10	6	<1	Asparagus	130	32	5	2	Brown Rice	150	55	33	18
Mushrooms	75	10	4	1	Radish	100	32	7	2	Ketchup	17	55	5	3
Cabbage	80	10	5	1	Mustard	5	32	1	<1	Apricots	120	57	9	5
Peanut Butter	55	14	5	6	Milk, skim	250	32	13	4	Potato	75	60	12	7
Peanut	28	14	6	1	Raspberries	150	32	8	3	Coca-Cola	250	60	26	16
Avocado	80	15	3	1	Ice cream	250	32	3	1	Fig (dried)	100	61	26	16
Zucchini	120	15	4	1	Pear	120	33	13	3	Beetroot	80	64	8	5
Cucumber	80	15	4	0	Apricot	120	34	9	3	Cantaloupe	120	65	6	4
Eggplant	100	15	6	2	Low Fat Milk	250	35	13	5	Sucrose	10	65	10	7
Tomato	100	15	4	1	Carrot	60	39	6	2	White rice	150	65	35	23
Celery	80	15	2	1	Plums	150	39	15	6	Couscous, boiled	150	65	35	23
Lettuce	100	15	3	1	Apple	120	40	16	6	Pineapple	120	66	10	6
Spinach	100	15	4	1	Orange	120	40	11	4	Sweet potato	130	70	17	12
Onion	10	15	1	<1	Strawberry	120	40	3	1	Crepe	30	71	7	5
Hazelnuts	28	15	5	<1	Pepper	2	40	1	<1	White bread	30	71	13	10
Red wine	150	15	4	<1	Apple Juice	250	40	30	12	Whole wheat bread	30	71	13	13
White wine	150	15	3	<1	Squash	80	41	30	8	Watermelon	120	72	6	4
Ginger	11	15	2	<1	Peach	120	42	11	5	Bagel	70	72	30	22
Yogurt, low fat	200	15	9	1	Beans, black-eyed	150	42	30	13	Goat milk	244	72	11	8
Soybean	190	16	56	9	Coconut	100	42	17	7	Rutabagas	385	72	33	24
Pistachios	28	18	8	1	Spaghetti	125	42	94	40	Popcorn	30	72	16	12
Walnut	28	20	4	1	Chocolate	28	43	16	7	Pumpkin	100	75	4	3
Cherries	100	20	16	5	Tagliatelle	125	44	90	40	Cornflakes	50	85	42	36
Lemon	60	20	5.5	1	Cranberry	110	45	8	1	Baguette	30	95	11	15
Pea	100	22	14	3	Endive	100	45	3	1	Glucose	10	100	10	10

Blue indicates low, orange indicates medium, and green indicates high GI or GL (www.glycemicindex.com) [1].

**Table 2:** Example of various diets’ composition for macronutrients with some examples of common foods associated with them. The low-GI diet highlighted in green is taken as a reference for a healthy diet.

	Low GL Diet	Regular Diet	Keto Diet	Modified Keto Diet	MCT Diet	Japanese Diet	Mediterranean Diet	Low GI Diet	Western Diet	High GI Diet	High GL Diet
**Carbohydrates**	45%	45–55%	5–10%	15%	5–10%	45–55%	50–60%	15–20%	50%	45%	55%
**Fat**	35%	20–35%	70–75%	55%	30% MCTs	20–35%	25–35%	60%	35%	35%	30%
					30% LCFA						
					10–15% others						
**Proteins**	20%	10–35%	20–25%	30%	20–25%	10–35%	5–25%	20–25%	15%	30%	15%
**Kcal**	2200	2200	2200	2200	2200	~80% of regular	2200	2200	~120% of regular	2200	2200
**Food**	low GL foods	Fresh food, low processed food	Low carbs food, High Fat, fish, meat, eggs, vegetables, fruits, nuts, berries…	Keto diet with increased amount of carbs	Keto diet enriched in MCT rich food such as coconut oil	Fish, Fruits, seasonal food, green tea, soy, rice (brown)…	Olive oil, fruits, vegetables and legumes, low amount of meat and fish, moderate wine	Low GI foods enriched, high non digestible fibers…	Junk foods, processed food with added sugar, saturated fats, high GI food…	High GI food, low non digestible fibers	high GL foods

The low-GI diet highlighted in green is taken as a reference for a healthy diet.

**Table 3:** Summary of human studies on diet effects on cognitive function in different neurological conditions.

Group	Diet	Method	Results	Limitation	Ref
Cognitive Healthy Elderly	No specific diet	Correlation between GI and cognitive score both assessed via questionnaire	Improved cognition in blood glucose regulation defect people	Different diets, background, food habits, medical history Questionnaire assessment of cognition only	[26,27,28,32]
Schoolchildren	Low GI breakfast vs. High GI breakfast vs. no breakfast Low GI/low GL vs. low GI/high GL vs. high GI/low GL vs. high GI/high GL	Cognitive test for learning and memory, accuracy and speed score, stress, hunger and thirst assessment	Low GI improves cognition and accuracy and decrease stress	Schoolchildren tested only during the morning for the GI breakfast. No effect measured after lunch or on a long time period.	[37,38,39,40,41,42,43,46]
Adults	No specific diet	Correlation between the GI of the diet and cognitive score	No effect	Only study, group compared to elderly No adults group with high GI diet	[47]
Epilepsy	KD, modified KD, low GI	Pediatric patients, number of seizure	50% decrease in the number of seizure	Observational studies, No interventional studies, no controlled diet, no longitudinal studies, no mechanistic studies, only hypothesis	[8,48]
Stroke	Vegetarian diets, Mediterranean diet High GI/GL diet	Stroke occurrence	Decreased risk of stroke with vegetarian diets Poor outcome following stroke with High GI/GL		[49,50,51,52,53,54,55]
Alzheimer’s disease	High GI Diet, Low GI, healthy diet, KD, MCT diet, Mediterranean diet	Post mortem brain analysis, memory test	High GI associated with accumulation of Aβ Healthy diet decrease Aβ, improves memory and verbal communication		[56,57,58,59]
Parkinson’s disease	Japanese diet	PD rate	Low PD rate		[60,61]
Autism Spectrum Disorder	High GI or low GI diet	Animal studies, social behavior analysis	High GI increase ASD phenotype while low GI improve social behavior		[62,63,64,65]
Depression and Anxiety	High GI/GL	Rate of disease in a population	Increased depression and anxiety rate		[66]
